# Exploring Nonresponse to Botulinum Toxin in Aesthetics: Narrative Review of Key Trigger Factors and Effective Management Strategies

**DOI:** 10.2196/69960

**Published:** 2025-04-24

**Authors:** George Kroumpouzos, Fernando Silikovich

**Affiliations:** 1 GK Dermatology PC, 541 Main St, Suite 320, South Weymouth, MA, 02190, United States, 1 617-501-1152, 1 781-812-2748; 2 Department of Dermatology, Warren Alpert Medical School at Brown University, Providence, RI, United States; 3 Concepto 4 Puntos Clinic, Buenos Aires, Argentina

**Keywords:** botulinum toxin, onabotulinumtoxinA, abobotulinumtoxinA, incobotulinumtoxinA, daxibotulinumtoxinA, aesthetic, cosmetic, trigger factor, neutralizing antibody, nonresponse, resistance, immune response, prevention, treatment, management

## Abstract

**Background:**

Nonresponse to botulinum toxin type A (BoNT-A) has been reported in both medical and aesthetic applications. Secondary nonresponse (SNR) occurs when BoNT-A is initially effective before failure commences at a later point. Most reported cases involve SNR in aesthetics. Several aspects of this complication remain elusive or controversial.

**Objective:**

We aimed to address unanswered questions regarding the prevalence and etiology of SNR. Additionally, we investigated the immunogenicity of BoNT-A formulations, mainly focusing on the development of neutralizing antibodies that hinder the toxin’s pharmacologic effects. Furthermore, we sought to examine the management strategies for SNR.

**Methods:**

The PubMed and Google Scholar databases were searched from inception for articles on nonresponse to BoNT-A therapy. Articles were evaluated based on their contribution to the field. Expert opinions and panel recommendations regarding management and data gaps were also included in the review.

**Results:**

There are limited data on SNR prevalence in aesthetic applications compared to therapeutic uses. Trigger factors of SNR include improper handling of BoNT-A; incorrect injection practices; and impurities present in the formulation, such as clostridial complexing proteins that may increase immunogenicity. Other contributing factors include infection; patient characteristics; and treatment parameters that encompass an increased frequency of BoNT-A injections (ie, <3 months apart), higher cumulative dosages, elevated treatment dosages, and booster injections (retreatment within 3 weeks of the initial injection). Neutralizing antibodies developed with first-generation formulations, such as onabotulinumtoxinA and abobotulinumtoxinA that contain clostridial proteins, but not with second-generation BoNT-As, such as incobotulinumtoxinA and daxibotulinumtoxinA, which lack these proteins. Among patients who developed SNR after using first-generation BoNT-A for aesthetic purposes, switching to incobotulinumtoxinA therapy did not result in the development of immune responses. Switching to a protein-free BoNT-A formulation such as incobotulinumtoxinA upon development of SNR has been advocated. To effectively manage SNR, it is crucial to minimize the identified trigger factors.

**Conclusions:**

Nonresponse to BoNT-A is gaining importance in aesthetic treatments. Considering the potential for immunogenicity is essential when selecting a BoNT-A formulation. Preventing SNR is crucial, given the lack of solid data on effective treatments.

## Introduction

Nonresponse or resistance to botulinum toxin type A (BoNT-A) has become an increasingly significant concern in the field of aesthetics, particularly since younger patients—who are increasingly opting for aesthetic procedures—accumulate greater total toxin doses over their lifetime. Resistance has been noted even with low BoNT-A doses in aesthetic treatments [1]. Primary nonresponse (PNR) to BoNT-A refers to individuals who show an innate insensitivity to the toxin upon initial exposure, without prior treatments or antibody (Ab) development. On the other hand, secondary nonresponse (SNR) occurs when BoNT-A is initially effective before failure commences at a later point. PNR is more commonly encountered in therapeutic applications [2], while most reported cases in aesthetic treatments involve SNR [3].

This review aims to address unanswered questions about the prevalence and etiology of SNR, with a particular focus on the immunogenicity of BoNT-A formulations and the development of neutralizing antibodies (NAbs) that hinder the toxin’s pharmacologic effects. We also explore management strategies for SNR.

## Methods

A narrative review was completed because a systematic review was not feasible due to the high heterogeneity among the articles on this topic. The PubMed and Google Scholar databases were searched from inception. Key search terms included “botulinum toxin,” “nonresponse OR nonresponsiveness OR resistance OR failure,” “aesthetic OR cosmetic,” “prevention,” and “management OR treatment OR intervention.” Separate searches were carried out for specific BoNT-A formulations using the following terms: “onabutulinum OR onabotulinumtoxinA” (onaBoNT-A), “abobutulinum OR abobotulinumtoxinA” (aboBoNT-A), “incobotulinum OR incobotulinumtoxinA” (incoBoNT-A), and “daxibotulinumtoxinA” (daxiBoNT-A). Additionally, reference lists of relevant articles were reviewed. Expert opinions and panel recommendations regarding management and data gaps were also included in the review.

## Results

### Principal Findings

We review the findings of publications relevant to the prevalence of SNR [4-8], etiology of nonresponse to BoNT-A [6,9-18], key trigger factors in SNR [2,6,11,14,15,19-26], BoNT-A formulations composition [4,8,11,23,27-52] and immunogenicity [1,3-6,10,11,15,17,18,28,31,35-38,44,48,49,53-67], insights into mechanisms of SNR [1,3,7,14,15,23,68,69], SNR management [1,3,6,7,14,23,25,26,46,66,70-81], and data gaps and limitations [3-6,10,11,14,15,26,28,40,49,63,82-84].

### Prevalence

The prevalence of SNR in therapeutic applications of BoNT-A varies among conditions treated and is often correlated with the toxin dose used. Detection of NAbs correlated to nonresponsiveness in therapeutic applications [4], with its global prevalence estimated at 0.3%‐27.6% [5]. Limited data exist regarding its prevalence in aesthetics, which is partly due to the diverse treatment approaches used and difficulties in quantifying the cosmetic effect [6]. In a recent survey among 673 Korean aesthetic providers, 53.9% reported experiencing BoNT-A resistance. Of those, 59% providers indicated the resistance rate as <1%, and 36% providers reported as approximately 1‐25% [7]. In the same study, 23.8% of respondents continued using the same product but at a higher dose when they suspected that a patient might be experiencing BoNT-A resistance. Therefore, the prevalence of resistance is likely underreported, as many providers are unaware and may solely increase the BoNT-A dose in subsequent sessions following a partial response [5,8].

### Etiology

#### Primary Nonresponse

PNR can be attributed to genetic variations that affect the toxin’s target molecules (neuronal receptors) or to a genetic predisposition to anti-BoNT antibodies formation due to different major histocompatibility complex types [9-11]. Genetic polymorphisms in immune response genes can influence how the body reacts to the toxin and can be involved in immunoresistance [12]. PNR has also been attributed to preexisting BoNT-A antibodies, possibly due to prior immunization against botulism [13,14].

#### Secondary Nonresponse

SNR to botulinum toxin (BoNT) is believed to be primarily due to the development of NAbs that hinder BoNT’s pharmacological effects [15]. This immune response can be influenced by epigenetic changes affecting the expression of genes involved in immune function, including those encoding for proteins interacting with BoNT [9,16]. The overall reactivity of an individual patient’s immune system–specifically, the ability of an antigen to stimulate an immune response–can be influenced by exogenous factors, such as environmental allergens. Some researchers consider this relevant, as most reported cases of complete SNR developed after multiple injection cycles [6,17,18]. In the series by Dressler et al [6], complete nonresponse occurred after 3, 5, 10, and 13 injection cycles, with treatment periods ranging from 16 to 65 months. However, more data on specific patient characteristics are needed.

### Key Trigger Factors in SNR

#### Toxin Handling and Injection Practice

Before attributing SNR to NAbs, it is important to consider other causes of nonresponse related to the handling of BoNT-A, such as improper dilution, prolonged storage under refrigeration, and interbatch variation [19-22]. Furthermore, SNR can also occur due to incorrect injection practices, which may involve insufficient dosing, targeting the wrong muscle, or using improper injection technique [19].

#### Toxin Purity

Impurities present in the BoNT-A formulations, such as clostridial complexing proteins, inactivated toxin, flagellin, and DNA contaminants, are believed to increase immunogenicity related to development of NAbs [23].

#### Vaccine

COVID-19 vaccination stimulates the immune system and may increase the risk of mounting an immune response against BoNT-A [24].

#### Patient Characteristics

Genetic differences in the control of immune responses indicate that patients exhibit variable speed and magnitude of immune reactions and patterns of NAb generation [14,25,26]. Some patients may have a specific predisposition to SNR; in one case, complete SNR occurred after just two injection sessions [6].

#### Treatment Parameters

Multiple treatment parameters affect BoNT-A immunogenicity. Due to it being a potential lifelong treatment, the prevalence of NAbs increases with chronic BoNT-A use [11]. The increased frequency of BoNT-A injections (ie, <3 months apart) is an essential trigger factor [14,15]. Other contributing factors include cumulative dosage, booster injections (retreatment within 3 weeks of the initial injection), high treatment dosage, and a patient’s immune responsiveness [2,23]. Notably, off-label aesthetic applications, such as masseter hypertrophy, whole face intradermal lifting, and body contouring require higher doses (ie, >100 international units of onaBoNT-A) and more frequent injections. Their increasing popularity may lead to increased prevalence of SNR and NAbs.

### BoNT-A Formulations Composition

All BoNT-A formulations contain the same 150-kDa core neurotoxin derived from the *Clostridium botulinum* Hall A strain [11,27,28]. The 150-kDa core neurotoxin contains a 100-kDa heavy chain and 50-kDa light chain, linked by a disulfide bond. BoNT-A formulations vary in purity, specific bioactivity, complexing proteins, and excipient content (Table 1), all of which can influence their potential to elicit an immune response.

**Table 1.** Characteristics and prevalence of NAb^a^ development and clinical nonresponsiveness of main first- and second-generation BoNT-A^b^ preparations.

Parameter	First-generation BoNT-A^c^		Second generation BoNT-A^d^	
	OnaBoNT-A^e^	AboBoNT-A^f^	IncoBoNT-A^g^	DaxiBoNT-A^h^^,^^i^
MW^j^ of bacterial protein, kDa [29-31]	~900	~300–500^k^	~150	~150; also, a 5-kDa stabilizing peptide (RTP004)
Accessory proteins present [32-34]	Yes	Yes	No	No
Total protein/vial [35-37]	5 ng/100 U	4.36 ng/500 U	0.6 ng/100 U	—^l^
Total core neurotoxin protein/100 MU^m^, ng [33,38]	0.73	0.65	0.44	—
Active neurotoxin protein/100 MU, ng [33,38,39]	0.44	0.44	0.44	0.45
Inactive neurotoxin protein/100 MU, ng^n^ [32,33]	0.29	0.21	0	—
Excipients^o^ [8,28,34,40]	HSA^p^, NaCl^q^	HSA, lactose	HSA, sucrose	RTP004 peptide, L-histidine, L-histidine-HCl monohydrate, polysorbate 20, trehalosedihydrate
Patients with NAbs in pivotal clinical trials, % [41-46]	0.0‐1.9	0.0‐3.6	0‐1.8	0
Patients with NAbs in real-world studies, % [4,47]	1.5‐7.0	1.7‐6.0	0.0‐0.5	—
Reports of clinical nonresponse [6,15,48]	Yes	Yes	No	No
Formulation notes [28,33,39,49-51]	Reduced protein load from original formulation (ie, reduced clostridial protein impurities and inactive BoNT-A)	Contains flagellin with potential adjuvant properties; contains complexing proteins	No complexing proteins; no inactive toxoids; no patients with SNR^r^	No complexing proteins; proprietary peptide claimed to aid in stability and delivery

a NAb: neutralizing antibody.

b BoNT-A: botulinum toxin type A.

c First-generation BoNT-A formulations contain core neurotoxins and accessory clostridial proteins.

d Second-generation BoNT-A formulations contain only the therapeutic neurotoxin without accessory proteins or other bacterial substances.

e onaBoNT-A: onabotulinumtoxinA.

f aboBoNT-A: abobotulinumtoxinA.

g incoBoNT-A: incobotulinumtoxinA.

h daxiBoNT-A: daxibotulinumtoxinA.

i Details on the formulation are not fully disclosed by the manufacturer.

j MW: molecular weight.

k Formulation is a mixture of species, with 300 and 500 kDa being the most common.

l Not available.

m MU: mouse unit

n Values for inactive neurotoxin are approximate and were estimated by Frevert et al [33], then reported by Kerscher et al [32].

o The excipient list is not exhaustive; additional peptides may be included in the diluent of BoNT-A formulations produced outside the United States.

p HSA: human serum albumin.

q NaCl: sodium chloride.

r SNR: secondary nonresponse.

First-generation BoNT-A formulations such as onaBoNT-A and aboBoNT-A contain pharmacologically unnecessary components such as complexing accessory clostridial proteins, inactive neurotoxin, clostridial DNA, and excipients (Table 1) that may increase the risk of immune response [8,23,28]. The accessory proteins assemble into a supramolecular structure that serves two main functions: protecting the core neurotoxin from low pH conditions when ingested orally and facilitating its absorption in the gastrointestinal tract [27]. The protective function is mediated via the nontoxic nonhemagglutinin protein and the absorption function via hemagglutinin proteins [11]. Importantly, the accessory proteins rapidly dissociate from the core neurotoxin at neutral pH [27,52].

Second-generation Bo-NT-As, such as incoBoNT-A and daxiBoNT-A lack accessory proteins because of their removal during purification [11]. DaxiBoNT-A contains an HIV-derived, highly charged peptide (RTP004) which, according to the manufacturer, binds noncovalently to the negatively charged BoNT-A molecule and stabilizes it by preventing protein aggregation [28]. Additionally, the peptide may bind to negatively charged neuronal surfaces, which could enhance the internalization of the neurotoxin. However, Martin et al [28] reported that the binding of RTP004 to negatively charged neuronal surfaces should not be considered selective, as all cell types are negatively charged due to the terminal sialic acid residues on surface glycoproteins.

### Immunogenicity of BoNT-A Formulations

#### Nonclinical Data

The total clostridial protein load—comprising accessory proteins and the core neurotoxin—and its composition determine the immunogenicity of each BoNT-A formulation [53]. Accessory proteins, especially hemagglutinin-1, can enhance the immune response as adjuvants [54,55]. Antibodies (Abs) against BoNT can be divided into NAbs, targeting the core neurotoxin, mainly the binding site on the heavy chain, and non-NAbs, typically targeting accessory proteins or clinically irrelevant sites on the core neurotoxin. While NAbs inhibit the clinical efficacy of BoNT, the non-NAbs do not impact its clinical effectiveness. In rabbit studies, immunization with the complete inactivated BoNT-A complex generated Abs with a stronger neutralizing effect than Abs induced by immunization with the core neurotoxin alone [54]. Accessory proteins may trigger increased production of inflammatory cytokines such as interleukin-6 and tumor necrosis factor-alpha and can bind to several nonneuronal cell types [55].

The total protein per vial of common BoNT-As is shown in Table 1 [35–37, 56 and 57]. IncoBoNT-A does not contain any inactive neurotoxin. In vivo studies indicate that onaBoNT-A injections generate antiBoNT-A Abs, with more frequent dosing leading to higher Ab levels [56]. In rabbits that received nine injections of onaBoNT-A or incoBoNT-A (at 2-8 week intervals), NAbs were detected in 20% of onaBoNT-A-treated animals, while none were detected in those treated with the accessory protein-free incoBoNT-A formulation [37]. AboBoNT-A contains less clostridial protein than onaBoNT-A, but its accessory proteins comprise up to 30% of the total clostridial protein content [11]. Importantly, the aboBoNT-A formulation also contains flagellin, which activates the toll-like receptor 5, thereby triggering an innate immune response [49].

The daxiBoNT-A formulation contains a proprietary, HIV-derived 5-kDa stabilizing peptide (RTP004) and polysorbate 20 [44]. This novel HIV-derived peptide is considered immunogenic [28]. As RTP004 binds to negatively charged areas on the surface of BoNT-A, it may create novel structures on the heavy or light chains of the core toxin that the immune system can recognize as neoepitopes. Polysorbate 20 may generate free radicals via auto-oxidization and can interact with other proteins in the formulation [28].

#### NAb Formation in Clinical Studies

BoNT-A treatment can trigger an adaptive immune response, especially with repeated injections, which may lead to NAb formation over time [11,57,58]. The rate of NAb development and occurrence of clinical resistance vary significantly by the BoNT-A formulation, particularly its protein content [59]. Table 1 shows the prevalence rates of NAbs in pivotal BoNT-A trials that supported approval by the US Food and Drug Administration (FDA). Pivotal onaBoNT-A and aboBoNT-A studies used the mouse protection assay (MPA), while incoBoNT-A studies used the mouse hemidiaphragm assay (MHDA), which is at least five times more sensitive than the MPA. Despite its greater sensitivity, the MHDA consistently revealed the lowest rates of NAb formation [10,59]. Analysis from phase 3 trials with daxiBoNT-A showed low rates of Ab formation to both daxiboNT-A and excipient RTP004 [45]. Treatment-related anti-daxiboNT-A and anti-RTP004 binding Abs were detected in 0.8% and 1.3% of subjects, respectively. No individual developed NAbs. Binding Abs were generally transient, of low titer (<1:200), and no individual had binding Abs to both daxiBoNT-A and RTP004. All individuals with treatment-induced binding Abs to daxiboNT-A or RTP004 showed clinical response at week 4 following each treatment cycle, indicating no impact on treatment efficacy. However, of the 2786 patients, 882 received two treatments and only 568 received three treatments. Therefore, the cumulative exposure and overall time frame for development of NAb-induced SNR may have been too short to draw robust conclusions.

The reported incidence rates of NAbs in product labeling are derived from short-term clinical trials and may not reflect real-world data, as repeated BoNT-A use can have cumulative effects over time [59]. Real-world studies with long-term follow-up have shown a reduction in NAbs in patients treated with incoBoNT-A [4,60,61]. A meta-analysis found that the prevalence of NAbs across indications is higher in patients treated with onaBoNT-A (around 1.5%) or aboBoNT-A (around 1.7%) compared to those receiving incoBoNT-A (0.5%) [4]. Although the overall prevalence of NAbs was low, there was a significantly higher rate of NAb development among patients who exhibit SNR [5]. Specifically, among patients with SNR, NAbs were observed in 32.5% patients treated with onaBoNT-A and 56.7% with aboBoNT-A. Notably, none of the patients who received incoBoNT-A developed SNR [4].

In an MHDA-based study, none of the toxin-naive patients who received incoBoNT-A treatment developed NAbs [62]. Furthermore, there have been no reported instances of clinical nonresponse among individuals who were toxin-naive at the time they received incoBoNT-A [10,62]. The formation of NAbs was rare in pivotal clinical trials, with only 9 out of more than 2600 patients treated with incoBoNT-A developing them [43]. A pooled data analysis from pivotal clinical studies on the aesthetic use of incoBoNT-A indicated no diminished treatment response due to the formation of NAbs [63]. Another study showed that switching to incoBoNT-A after SNR with another BoNT-A formulation enabled patients to regain responsiveness to treatment, with NAbs developing only in two patients previously treated with aboBoNT-A [62].

### SNR and NAb in Aesthetic Studies

Case studies of BoNT-A use for aesthetic purposes demonstrated both SNR and NAb development over time with onaBoNT-A and aboBoNT-A [6,15,48,63]. In general, prevalences of NAb development and SNR are lower in aesthetic indications (overall NAb rate estimated at 0.2%‐0.4%) [5], which may reflect the lower doses employed and minimal long-term data [15,49,64]. Thirteen cases of NAb-related SNR emerging during aesthetic BoNT-A treatments [1,3,6,15,18,65,66] were identified in case reports or series. Key observations of this review are presented in Textbox 1. Complete SNR is usually preceded by partial SNR in the patient [6,17,18]. Complete SNR usually occurs after more than two injection series [6,17]. It can occur as long as after 5 years of treatment [6,17]. In a small sample study, 30% of patients who did not respond to onaBoNT-A cosmetic treatments responded when switched to incoBoNT-A therapy, which did not provoke immune responses [69].

Textbox 1. **Key observations in reports detailing secondary nonresponse (SNR) to botulinum toxin type A (BoNT-A) aesthetic treatment.**

- Seven reports detailing a total of 13 cases [1,3,6,15,18,65,66]
- Patients initially or exclusively received onabotulinumtoxinA (onaBoNT-A) or aboBoNT-A (aboBoNT-A)
- SNR developed even after low BoNT-A doses [1,6,65]
- Regular repeated treatments before development of SNR, with clear signs of increasing dosages and shortening intervals between treatments
- Partial SNR observed as early as 2nd injection cycle [6] and complete SNR as early as 1st cycle [15]; partial SNR usually preceded complete SNR [6]
- Duration of therapy before natural antibody (NAb) detection variable (2‐72 months) [15,65]
- Systematic testing for detecting NAb formation was infrequent and, in most cases, it was unclear when NAb formation first occurred
- No cases of NAb-related SNR were reported with exclusive incobotulinumtoxinA (incoBoNT-A) use
- Four patients were switched to incoBoNT-A after partial or complete SNR [1,6,15,65; this switch showed no treatment effect
- Switch to incoBoNT-A associated with downward trend in NAb titer [66]
- After SNR, injection of botulinum toxin type B (BoNT-B) showed a normal therapeutic effect [1,6]

After switching from BoNT-A to BoNT-B, NAbs to the latter may develop because the heavy chains of BoNT-A and BoNT-B have a 30% structural homology [26]. Patients who initially respond to BoNT-B after developing SNR to BoNT-A are likely to eventually develop SNR to BoNT-B as well [67,68].

## Discussion

### Insights Into Mechanisms of SNR

Retrospective studies suggest an association between higher protein exposure and increased risk of Ab formation [14,70,71]. The precise mechanisms leading to resistance are still unknown, as the pure 150-kDa neurotoxin has low immunogenicity without any known associated pattern recognition receptors or toll-like receptors on dendritic cells. Park et al [23] suggested that when adjuvants in the BoNT formulation are injected alongside the 150-kDa neurotoxin, they can activate dendritic cells that may internalize the neurotoxin and present it to T-helper lymphocytes, resulting in NAb formation. Exogenous factors such as environmental allergens (eg, COVID-19 vaccine) may prime NAbs [72,73]. Specific immune system activation by a wasp sting was proposed as a contributing factor for BoNT-A Ab formation [74].

Alternate explanations for resistance to BoNT-A include muscle injection fibrosis, BoNT receptor downregulation, dynamic line depth worsening, and interactions with drugs like aminoglycosides and quinolones [3]. Intradermal injections are thought to carry a higher risk of developing resistance to BoNT-A compared to intramuscular injections, as the dermis is rich in antigen-presenting dendritic cells [5,7]. A phenomenon of decreased responsiveness after many years of BoNT-A therapy, known as tachyphylaxis, has been reported [1]. In such cases, the clinical effect is mitigating despite the absence of NAbs. Nevertheless, it is still uncertain whether this phenomenon has an immunologic basis and whether low-titer or poorly binding antibodies might play a role.

### SNR Management: Early Diagnosis

Early diagnosis is crucial, particularly as an increase in NAb formation must be addressed promptly. A patient’s aesthetic journey, especially a need for increasing BoNT-A doses and more frequent treatments, should alert the provider of possible SNR. Accurately detecting and quantifying NAbs supports the diagnosis. Structural assays such as ELISA and immunoprecipitation assays are sensitive for detecting BoNT Abs, but do not discriminate between NAbs and non-neutralizing Abs [14,15,23]. Bioassays such as the MPA or MHDA use animal models to identify NAbs. The MHDA, the only assay approved by the FDA, uses ex vivo testing for NAbs [14].

Most clinicians do not have access to the above assays and use clinical resistance tests to confirm the diagnosis of SNR [1,14]. One such test is the unilateral brow injection, which involves injecting a standard amount of BoNT-A, such as 20IU onaBoNT-A, into the right (by convention) medial eyebrow [14]. After allowing sufficient time for the toxin to take effect (typically 1‐3 weeks), the frowning facial expression is evaluated. Since nearly all individuals usually frown symmetrically, asymmetric frowning indicates responsiveness to the injected BoNT-A that has weakened the right corrugator or procerus muscles. In contrast, symmetric frowning indicates that the injected muscles were not weakened; therefore, the patient is likely resistant to that specific type of BoNT-A.

### Preventive Measures

Several authors have advocated for using a highly purified toxin that demonstrates the least immunogenicity, such as incoBoNT-A [15,23]. This is especially important in large-dose injections and while treating younger patients who will accumulate higher lifetime doses [23]. Most experts recommend using the smallest BoNT-A dose that achieves the desired clinical effect, avoiding booster injections, and waiting at least 3 months between treatments [6,7,15]. Regarding maximum dose, 56.5% of aesthetic providers responded that BoNT-A dose should be limited to <100 IU per day, and 97.3% reported using <300 IU in total [7]. Such total doses are unlikely for wrinkle reduction but are possible with some off-label indications such as muscle size reduction. In body indications, higher doses of BoNT-A are injected, increasing a patient’s exposure to foreign proteins and their risk of NAB formation. Consequently, it is advisable to use a highly purified BoNT-A when treating body indications.

Increasing the efficacy and longevity of outcomes of BoNT-A treatments leads to decreased frequency of such treatments, which can help prevent resistance. Several authors recommend using toxins that offer improved longevity for cosmetic results, such as daxiBoNT-A [46]. In two of three randomized controlled trials, coadministration with oral zinc supplementation enhanced the longevity of BoNT-A outcomes [75-77]; however, the available data are limited. Hyaluronidase is a known tissue permeability modifier that increases the dispersion of drugs [78]. In a small pilot study on axillary hyperhidrosis, the coadministration of BoNT-A with hyaluronidase allowed for a reduction in the BoNT-A dose needed to achieve a similar effect compared to BoNT-A injections administered alone [79]. Notably, in one patient, the right side of the forehead–treated with both BoNT-A and hyaluronidase–exhibited a larger area of effect than the left side, which received only BoNT-A, across all postinjection evaluations. The authors suggested that the reduced dose of BoNT-A required when used alongside hyaluronidase may be attributed to the enhanced dispersion of the toxin facilitated by hyaluronidase. This approach could help avoid the use of high toxin doses that may lead to nonresponsiveness over time. However, more data are needed to confirm these findings.

### Treatment

Switching to a highly purified toxin such as incoBoNT-A once partial SNR is noted, has been advocated [14,23,66], especially as this was associated with a downward trend in NAb titers [66,80]. This switch was associated with clinical response in a study of patients with cervical dystonia [81,82] and another involving onaBoNT-A cosmetic treatments [69]. Nevertheless, in our review of aesthetic treatments (Textbox 1), this switch was not associated with short-term SNR resolution [3,6,15,65]. Longer follow-up is required for aesthetic applications in patients with SNR switching to incoBoNT-A. A switch to daxiBoNT-A may also be considered given its low immunogenicity in limited studies [46], but more data is required. The first author successfully used a short course of low-dose oral methotrexate immediately before BoNT-A injection to mitigate an immune response leading to further reduction of clinical efficacy in patients who experienced partial SNR. Patients with prior complete or partial SNR to onaBoNT-A may benefit from anti-calcitonin gene-related peptide monoclonal Ab therapy [83].

For complete nonresponse, many experts advise offering a 12- to 18-month “drug holiday,” and then resuming with a highly purified toxin. This suggestion is based on the medical applications of BoNT-A [67,84] and aims to normalize NAb levels before administering BoNT-A again. The duration of the “drug holiday” should be determined by measuring NAb levels. However, other experts argue against offering a “drug holiday,” noting that switching to incoBoNT-A results in most patients’ NAb titers returning to negative, similar to those who stopped receiving BoNT-A treatment altogether [80]. Moreover, switching to incoBoNT-A may be the only option for patients whose NAb titers take longer to become negative [67,80].

Switching to a different BoNT serotype, such as type B (BoNT-B), has been attempted. For cervical dystonia, switching to BoNT-B (rimabotulinumtoxinB), was beneficial [62]. In two patients reviewed here, after SNR developed, injection of BoNT-B showed a normal therapeutic effect [1,6]. However, patients who switched from BoNT-A to BoNT-B after developing NAbs and SNR may subsequently develop NAbs to BoNT-B due to the 30% structural homology in the heavy chains of BoNT-A and BoNT-B [26]. Several studies have demonstrated that patients who initially respond to BoNT-B after developing SNR to BoNT-A are likely to eventually develop SNR to BoNT-B as well [67,68]. Additionally, injecting BoNT-B, an off-label toxin in aesthetics presents challenges, including suboptimal longevity and adverse effects such as an intense stinging sensation on injection [85,86].

### Data Gaps and Limitations

Aesthetic studies on NAb formation and SNR have been limited and have primarily focused on approved indications [4,6,63,87], while off-label applications involving higher BoNT-A doses have not been investigated. Additionally, the follow-up periods in these studies were relatively short (4-16 months), although NAbs usually develop over a more extended period, often spanning several years [5,40]. The frequency of NAb formation and SNR in real-world aesthetic practice may be higher than published estimates [5], likely due to extensive off-label use and the lack of a commercially available test for measuring NAb levels [11].

Detecting NAbs depends on the specific assay used, as there can be significant variability in sensitivity and specificity [10,11]. It also depends on the assay methodology, handling, and timing of collection of samples, and concurrent use of medications. Although the MHDA is the most sensitive bioassay, it is semiquantitative and not widely available. However, this assay has raised concerns about false-positive results and may detect subclinical Ab titers that do not result in treatment failure [1,14,15]. A quantitative, FDA-approved, commercially available assay to measure NAbs is needed to study the temporal variations in Ab titers [11]. This limitation prevents robust conclusions regarding the relationship of NAbs with nonresponsiveness. A lack of studies comparing BoNT-A formulations with a standardized NAb assay hinders reliable comparisons. Finally, it remains unclear to what extent the accessory proteins, inactive neurotoxin, and excipients may trigger the immune system, especially since the time frame for developing Ab-mediated SNR was short in most studies (ie, up to three injection cycles) [28]. This hampers our ability to draw firm conclusions regarding the excipients’ impact on the BoNT-A formulation’s immunogenicity.

A key uncertainty involves the relationship between NAbs and SNR [14]. Some patients with detectable NAbs retain their clinical responsiveness, while others without detectable NAbs have been nonresponsive to BoNT-As [14,49]. This indicates that there is no absolute correlation between NAb detection and nonresponse [88], and no established threshold for NAb titer reliably predicts clinical resistance to BoNT-A [3]. However, a correlation between responsiveness and NAb titers has been proposed [6,89]. Further complicating patient responses, variations in target binding site and binding affinity result in anti-BoNT-A Abs with variable neutralizing effects [10,26]. These observations highlight the complexity of BoNT-A immunogenicity and the variability in individual patient responses [14].

### Conclusions

Nonresponse to BoNT-A is becoming increasingly important in aesthetics, particularly as many patients undergo lifelong treatments. Preventing SNR is crucial given the lack of solid data on effective treatments. When choosing a BoNT-A formulation, considering the potential for immunogenicity is essential. Aesthetic providers should perform comprehensive clinical assessments, inform patients about the associated risks, and develop strategies to minimize immunogenicity in their treatment protocols.

## ABBREVIATIONS

Ab: antibody
AboBoNT-A: abobotulinumtoxinA
BoNT-A: botulinum toxin type A
BoNT-B: botulinum toxin type B
DaxiBoNT-A: daxibotulinumtoxinA
FDA: Food and Drug Administration
IncoBoNT-A: incobotulinumtoxinA
MHDA: mouse hemidiaphragm assay
MPA: mouse protection assay
NAb: neutralizing antibodies
OnaBoNT-A: onabotulinumtoxinA
PNR: primary nonresponse
SNR: secondary nonresponse
